# Emergency trauma admissions in the oldest-old: short- and long-term mortality and the role of frailty in a Turkish national cohort of centenarians

**DOI:** 10.1007/s00068-026-03137-0

**Published:** 2026-03-09

**Authors:** Merve Güner, Yavuz Şahbat, Sinem Bayram, Serdar Ceylan, Rıfat Bozkuş, Naim Ata, Mustafa Cankurtaran, Şuayip Birinci

**Affiliations:** 1 Division of Geriatric Medicine, Department of Internal Medicine, Faculty of Medicine, Istinye University, Istanbul, Türkiye; 2 Department of Orthopedics and Traumatology, Istinye University Bahçeşehir Liv Hospital, Istanbul, Türkiye; 3 Ministry of Health, Health Information Systems, Ankara, Türkiye; 4 Ministry of Health, Division of Geriatric Medicine, Department of Internal Medicine, Antalya City Hospital, Antalya, Türkiye; 5 Ministry of Health, Department of Internal Medicine, Ankara Etlik City Hospital, Ankara, Türkiye; 6 Ministry of Health, General Directorate of Health Information Systems, Ankara, Türkiye; 7 Division of Geriatric Medicine, Department of Internal Medicine, Faculty of Medicine, Hacettepe University, Ankara, Türkiye; 8 Ministry of Health, Deputy Minister, Ankara, Türkiye

**Keywords:** Centenarian, Orthopedic trauma, Hip fracture, Frailty, Longevity

## Abstract

**Introduction:**

This study aimed to evaluate the impact of orthopedic trauma on mortality among centenarians presenting to the emergency department, to examine the relationship between frailty and longevity, and to provide insights into post-trauma survival in long-lived individuals, as well as the role of frailty mechanisms.

**Methods:**

Data were retrieved from the National Personal Health Record System (NPHRS) of Türkiye. The study included patients aged ≥ 100 years who presented to emergency departments with trauma between January 2016 and July 2024 across all levels of healthcare institutions. Frailty was assessed using the Canadian Institute for Health Information Hospital Frailty Risk Measure (CIHI-HFRM) and 5-factor modified frailty index (mFI-5) based on ICD-10 codes. Survival status was determined retrospectively from national death registry.

**Results:**

A total of 1,180 patients were included, with a mean age of 101.3 ± 3.9 years. Hip fractures were the most frequent presentation, accounting for more than 40% of all cases. Overall, 906 patients (76.8%) died, with a median survival of 7.2 months (range: 0.1–51.1 months). Higher frailty scores according to the CIHI-HFRM were paradoxically associated with reduced mortality across follow-up intervals: 30-day (HR = 0.026, 95% CI: 0.006–0.118, *p* < 0.001), 90-day (HR = 0.091, 95% CI: 0.031–0.271, *p* < 0.001), and one-year mortality (HR = 0.084, 95% CI: 0.036–0.195, *p* < 0.001).

**Conclusion:**

Frailty, as defined by CIHI-HFRM and mFI-5, was not a risk factor for mortality at 30-, 90-, or 1-year follow-up; rather, it appeared protective. This paradoxical finding highlights the need to reconsider how frailty is conceptualized and measured in centenarians.

**Level of evidence:**

3.

**Supplementary Information:**

The online version contains supplementary material available at 10.1007/s00068-026-03137-0.

## Abstract (fr)

This study examined the short- and long-term mortality in centenarians admitted to the emergency department with an orthopedic trauma between 2016 and 2024 via a national cohort. It was revealed that the most frequent trauma is hip fracture, and the presence of hip fracture is an independent predictor of mortality. Additionally, factors at the facility level played a role, indicating that the availability of higher resources and healthcare could significantly impact survival rates after injuries, especially at very advanced ages. In contrast to expectations, frailty, as defined by electronic health records, was associated with improved survival. This paradox suggests that frailty measurement methods cannot be able to detect biological and psychosocial resilience mechanisms, and highlights the need to reconsider how frailty is conceptualized and measured in centenarians.

## Introduction

Increased lifespan has become an important health indicator in modern societies. Longevity is related not only to chronological age but also to health status, functional capacity, and disease resistance [1]. Longevity can be explained by a combination of genetic factors, low levels of inflammation, effective metabolic adaptations, and environmental support [2–5]. These mechanisms enable older individuals to develop resistance to many chronic diseases and extend their life spans. Centenarians represent individuals who have reached the limits of human life and constitute a unique group, both clinically and epidemiologically, due to their exceptional longevity [6]. Although expected mortality rates after trauma and illness are generally high in these individuals, in some cases, results inconsistent with frailty criteria may be observed. This situation suggests that the biological and functional reserves of centenarians differ from the risk predicted by frailty indices [7, 8].

Frailty is characterized by a decrease in homeostatic reserves, an inadequate stress response, and impaired coordination between physiological systems in older individuals; this leads to reduced tolerance to trauma or acute illness and an increased risk of mortality [9]. However, the longevity observed in centenarians does not always align with the classic profile of frailty. In these individuals, several biological mechanisms may mitigate the impact of frailty factors on mortality [10, 11]. For example, genetic advantages, including the presence of alleles associated with longevity, regulation of the inflammatory response, and resistance to chronic inflammation, control of so-called “inflammaging” and “anti-inflammaging”, as well as metabolic flexibility, adaptive capacity in glucose and lipid metabolism, and resilient cardiovascular systems, optimize the post-traumatic stress response [12, 13]. Additionally, some centenarians have strengthened both their physiological and psychological reserves through healthy lifestyle habits and strong social support systems from an early age [14]. This combination allows them to survive beyond expected mortality after trauma or illness, even if their frailty scores are high.

We hypothesized that higher frailty would be associated with increased mortality risk across follow-up periods and that hip fracture and lower-level care settings would be associated with poorer survival. Therefore, we aimed to characterize the distribution of orthopedic trauma among centenarians presenting to the emergency department in a nationwide cohort and to examine the association between frailty on 30-day, 90-day, and 1-year survivals, examine the relationship between frailty and longevity, and contribute to understanding the post-trauma survival of long-lived individuals and the role of frailty mechanisms.

## Materials and methods

### Study design and population

This retrospective cohort study used a nationwide electronic health database to identify trauma-related emergency department admissions among centenarian patients (≥ 100 years) in Türkiye between January 1, 2016 and July 31, 2024. The study was designed and reported in accordance with the Declaration of Helsinki. All data were anonymized prior to analysis, and the data use and analysis protocol were signed by the authors and approved by the relevant authorities. Demographic characteristics (age, sex), comorbid conditions, as recorded by ICD-10 codes in National Personal Health Record System (NPHRS), index diagnosis codes, and facility type/level were extracted. Survival status was assessed at 30 days, 90 days, and 1 year following the index emergency department encounter based on mortality information from the national death registry.

### National personal health record system

This study received approval from the Ministry of Health and the relevant institutional review board and was conducted in accordance with the Declaration of Helsinki. Data were obtained from NPHRS of Türkiye, an integrated, nationwide, real-time electronic health database established on January 1, 2015, and maintained by the Ministry of Health. The NPHRS contains comprehensive information from all public, university, and private healthcare facilities in Türkiye, including demographic characteristics, confirmed and suspected diagnoses, ICD-10 coding, medication and surgical details, laboratory and imaging results, and hospital admission and discharge dates.

NPHRS includes nationwide coverage across all healthcare levels and institution types. For the present study, the cohort consisted predominantly of admissions from secondary- and tertiary-level hospitals, with a minor proportion originating from primary care centers (as recorded in NPHRS facility-level metadata). Institution type (public/university/private) and healthcare level (primary/secondary/tertiary) were extracted for each index encounter and used to characterize the setting.

### Electronic query and case identification

Eligible patients were identified through a structured electronic query within NPHRS. The query applied the criterion according to age, patients aged ≥ 100 years at the index emergency department visit; admission type as emergency department visit between January 2016 and July 2024. Diagnosis criterion was established by the presence of ICD-10 codes consistent with traumatic orthopedic injury at the index encounter by the researcher (M.G and Y.S.). The electronic query was performed by the researcher who was an experienced data analyst (S.B.) and executed via the authorized NPHRS data access interface. Extracted records underwent a predefined quality-control workflow: removal of duplicate encounters, verification of key identifiers and encounter dates, and screening for missing or internally inconsistent fields. Another two researcher (R.B. and N.A.) independently reviewed two random subsets of records and cross-checked the eligibility logic to confirm the consistency of the extraction and cleaning steps. Records with incomplete demographic information, missing follow-up status, or inconsistent coding were excluded.

### Inclusion and exclusion criteria

We included patients aged ≥ 100 years admitted to the emergency department with a history of trauma and an ICD-10 diagnosis compatible with orthopedic trauma. Exclusion criteria were defined as incomplete demographic or follow-up data; multiple trauma requiring intensive care admission as recorded in NPHRS; open fractures and pathological fractures secondary to metastatic disease or underlying rheumatologic conditions. Of 1,228 eligible patients, 48 were excluded due to missing data, leaving 1,180 patients for the final analysis.

### Assessment of frailty

The Canadian Institute for Health Information Hospital Frailty Risk Measurement (CIHI HFRM) employs a cumulative deficit approach, which involves assessing an individual’s risk of frailty by accumulating deficits [15].

The CIHI HFRM uses a list of 36 frailty deficit categories, each associated with ICD-10 diagnosis codes (Supplementary Table 1). These categories include issues related to morbidity, functional status, sensory loss, cognition, and mood. A patient’s frailty risk over a two-year period is determined by counting the number of deficits present from their index discharge date in the reporting year; for our study, this date was accepted as the admission date to the emergency department. To obtain a continuous score, the total deficits for each patient are divided by 36, the maximum possible number of deficits, producing a value between 0 and 1. Patients are classified into eight risk groups based on the total number of deficits, from lowest risk (group 1) to highest risk (group 8): Risk group 1 with 0 or 1 deficit, Risk group 2 with 2 or 3 deficits, Risk group 3 with 4 or 5 deficits, Risk group 4 with 6 or 7 deficits, Risk group 5 with 8 or 9 deficits, Risk group 6 with 10 to 12 deficits, Risk group 7 with 13 to 15 deficits, and Risk group 8 with 16 or more deficits (Table 1).

**Table 1:** CIHI Hospital Frailty Risk Measure (HFRM) risk groups and corresponding deficit counts

Risk Group (Severity)	Continuous CIHI HFRM (Range)	Total Number of Deficits per Patient	Frailty Risk Status
1	0–0.028	0–1	Not at risk
2	0.056–0.083	2–3	
3	0.111–0.139	4–5	
4	0.167–0.194	6–7	At risk
5	0.222–0.250	8–9	
6	0.278–0.333	10–12	
7	0.361–0.417	13–15	
8	≥ 0.444	16+	

Frailty was additionally quantified using the 5-factor modified frailty index (mFI-5), which can be derived from routinely collected administrative diagnosis data. The mFI-5 consists of five deficits including diabetes mellitus, congestive heart failure, hypertension requiring medication, chronic obstructive pulmonary disease, and functional dependence (non-independent functional status) and for each patient, these deficits were identified from ICD-10 diagnosis codes. The mFI-5 score was calculated as the sum of deficits divided by 5, yielding a value between 0 and 1, and higher values indicate greater frailty burden [16].

### Statistical analysis

The statistical analysis was performed using SPSS software version 26.0. The variables were assessed using visual (histograms, probability plots) and analytical (Kolmogorov-Smirnov test) methods to determine whether they were normally distributed. Descriptive analyses are presented using percentages for categorical variables, mean ± standard deviations (SD) for normally distributed variables, and median [interquartile range (IQR)] for non-normally distributed variables. The chi-square test or Fisher’s exact test was used to compare categorical variables. The Mann–Whitney U test or Student’s t-test was used to compare continuous variables based on their distributions. Cox proportional hazards analysis was used to evaluate the effects of independent variables on 30-day, 90-day, and 1-year survival. First, univariate analysis was performed, and the variables found to be significant (gender, age, trauma site, hospital volume, and frailty) were included in the multivariate models. In Cox regression, CIHI-HFRM was used as a continuous predictor and scaled to report hazard ratios for each 0.1 increase (CIHI×10) and for each additional deficit (CIHI×36). The primary Cox regression models were repeated, replacing CIHI-HFRM with mFI-5, to assess the robustness of the frailty–mortality association. The results of the Cox analysis were presented as hazard ratios (HRs) with 95% Confidence Intervals (CIs). The Kaplan-Meier survival curves were demonstrated for frailty groups. *p* < 0.05 was considered the cutoff for statistical significance.

## Results

**Fig. 1:**
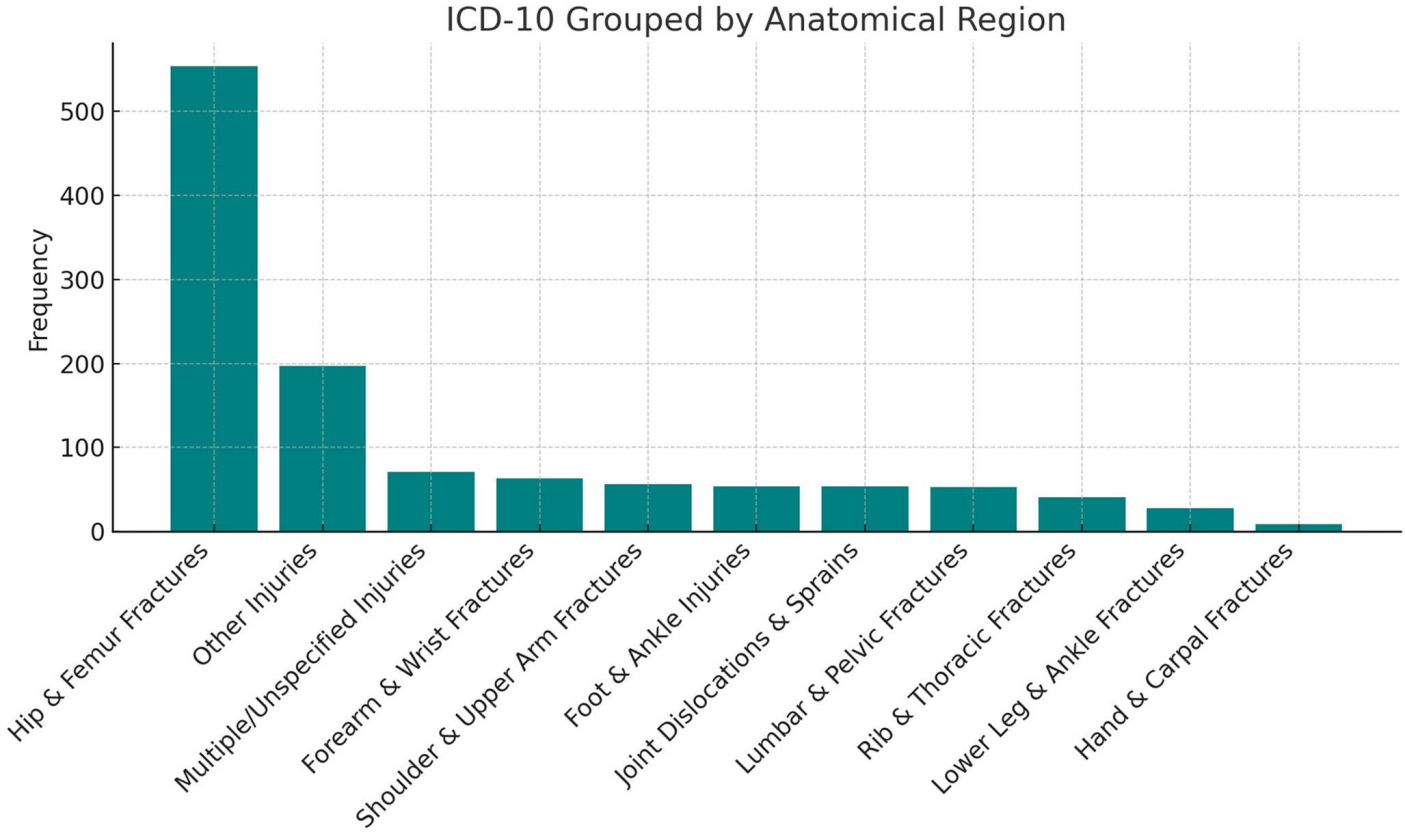
The distribution of the trauma sites according to the anatomic region (Bar chart)

The study included 1,180 patients with a mean age of 101.3 ± 3.9 years. Of these, 85.9% were female. Most patients were managed at secondary-level hospitals (62.5%) or tertiary-level hospitals (37.5%), with only a negligible proportion recorded in primary care (Table 2). Injury distribution is presented in Fig. 1. Hip fractures were the most common presentation to the emergency department, accounting for more than 40% of cases, followed by fractures of the upper extremities, specifically the radius, humerus, and rib fractures. Overall, 906 patients (76.8%) died during follow-up, with a median overall survival of 7.2 months (range: 0.1–51.1 months). Early mortality occurred in 15 patients within 24 h and 31 patients within 72 h of emergency department admission (Table 2).

**Table 2:** Demographic characteristics, trauma localization, and mortality outcomes of centenarian patients (*n* = 1180)

	*n* = 1180
Age, years	101.0 [100–103]
**Gender**	
Male	166 (14.1%)
Female	1014 (85.9%)
**Healthcare facility**	
Primary Care	1 (0.1%)
Secondary Center	737 (62.5%)
Tertiary Center	442 (37.5%)
Overall Mortality Rate	906 (76.8%)
Overall Survival, in months	7.2 [1.3–21.2]
One-month mortality	188 (15.9%)
Three-month mortality	333 (28.2%)
One-year mortality	545 (46.2)

Values are presented as n (%) for categorical variables and median [IQR] for continuous variables, unless otherwise stated. Age represents age at index emergency department admission. Overall mortality refers to death during the total follow-up period. One-month, three-month, and one-year mortality were defined as death within 30, 90, and 365 days after index admission, respectively. Overall survival time is reported as median [IQR] for the entire cohort

Compared with the non-survivor group, survivors had significantly higher rates of anemia, arthritis, asthma, coronary artery disease, diabetes mellitus, and vertigo (all *p* < 0.001). In contrast, no significant differences were observed for sex or other comorbidities evaluated (Table 3). Furthermore, the distribution of 1-year mortality across CIHI-HFRM risk groups showed lower mortality in higher deficit-burden categories (Supplementary Table S2).

**Table 3:** Comparison of comorbidities and clinical characteristics between survivor and non-survivor groups

	Survivor (*n* = 274)	Non-survivor (*n* = 906)	*p*
Gender, female	244 (89.1)	770 (85.0)	0.090
Anemia	120 (43.8)	288 (31.8)	< 0.001
Depression	163 (59.5)	506 (55.8)	0.29
Dysphagia	2 (0.7)	16 (1.8)	0.28
Arthritis	184 (67.4)	457 (50.4)	< 0.001
Asthma	99 (36.1)	228 (25.2)	< 0.001
COPD	78 (28.5)	258 (28.5)	1.0
HF	61 (22.3)	227 (25.1)	0.35
CAD	107 (39.1)	232 (25.6)	< 0.001
DM	104 (38.0)	243 (26.8)	< 0.001
Vertigo	163	388	< 0.001
Malignancy	-	-	-
Arrhythmia	70	192	0.13
Cerebrovascular event	48 (17.5)	123 (13.6)	0.10
Dementia	135 (49.3)	480 (53.0)	0.28
Delirium	20 (7.3)	88 (9.7)	0.23

Values are presented as n (%). Non-survivors were patients who died during follow-up, and survivors were those alive at the end of the follow-up period. Categorical variables were compared using the chi-square test or Fisher’s exact test when appropriate

COPD, chronic obstructive pulmonary disease; HF, heart failure; CAD, coronary artery disease; DM, diabetes mellitus

**Fig. 2:**
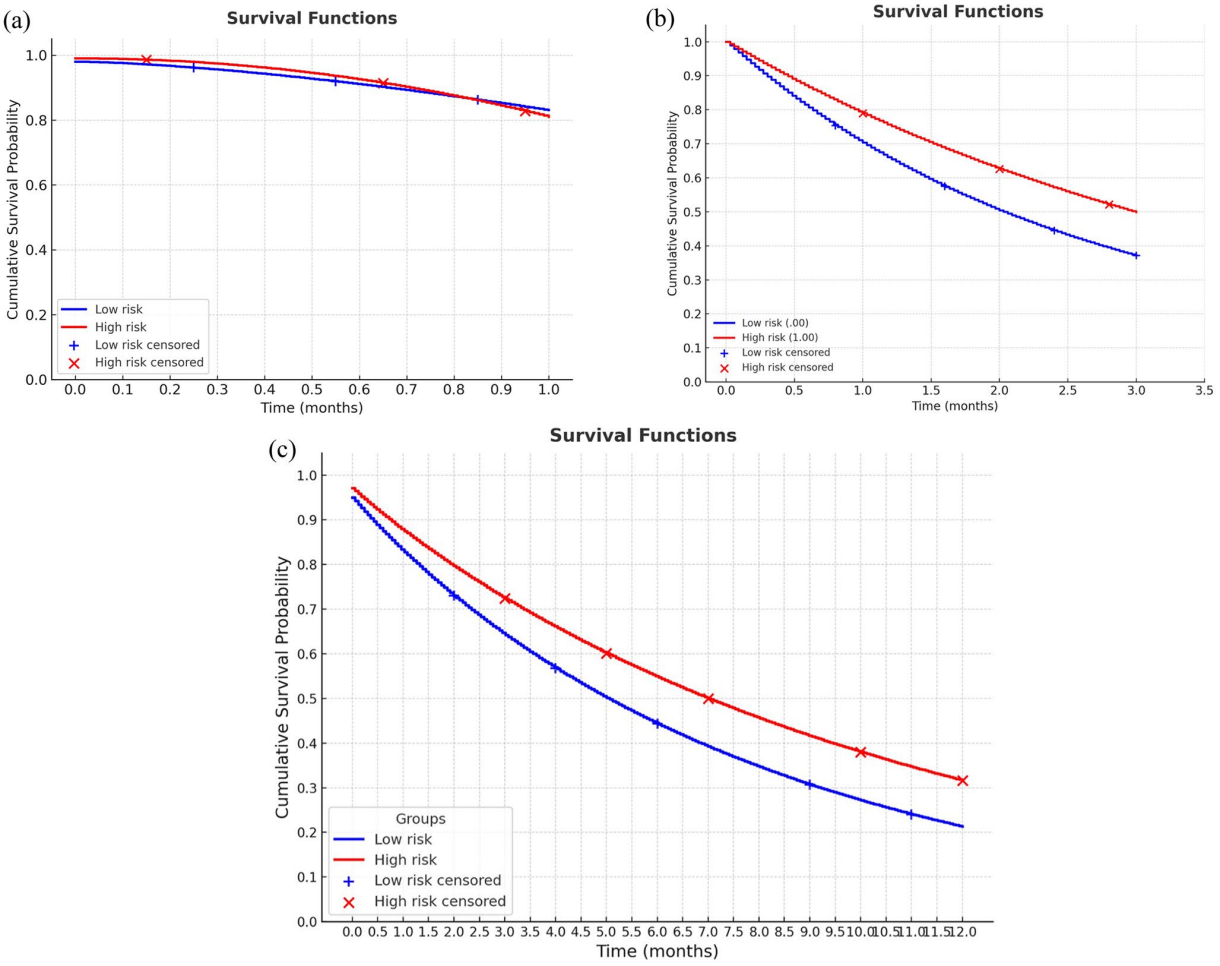
The survival curves of centenarians according to their frailty status at (**a**) 1 month and (**b**) 3 months and (**c**) 1 year

In the Cox regression analyses (Table 4), hip fracture was the strongest independent predictor of mortality at all time points. Patients with hip fractures had nearly a three-fold higher risk of 30-day mortality (HR = 2.962, 95% CI: 2.162–4.057, *p* < 0.001), with the excess risk persisting at 90 days (HR = 2.529, 95% CI: 2.014–3.176, *p* < 0.001) and one year (HR = 2.080, 95% CI: 1.750–2.472, *p* < 0.001). On the other hand, higher frailty scores according to the CIHI index were associated with lower mortality across all follow-up periods: 30-day (HR = 0.026, 95% CI: 0.006–0.118, *p* < 0.001), 90-day (HR = 0.091, 95% CI: 0.031–0.271, *p* < 0.001), and one-year mortality (HR = 0.084, 95% CI: 0.036–0.195, *p* < 0.001). Male gender was not significantly associated with early mortality (within 30 days and 90 days); however, it predicted higher one-year mortality (HR = 1.269, 95% CI: 1.012–1.592, *p* = 0.039). Age at admission was not related to short-term outcomes but was independently associated with lower one-year survival (HR = 0.971, 95% CI: 0.945–0.997, *p* = 0.027). Finally, treatment at higher-level healthcare facilities (tertiary care) was consistently protective, significantly reducing mortality compared with primary or secondary care (all *p* < 0.001). In another model using mFI-5, frailty remained inversely associated with mortality at 30 days (HR 0.782, 95% CI 0.683–0.896; *p* < 0.001), 90 days (HR 0.899, 95% CI 0.816–0.990; *p* = 0.031), and 1 year (HR 0.906, 95% CI 0.840–0.976; *p* = 0.010). The Kaplan-Meier survival curves for one month, three months, and one year are demonstrated in Fig. 2.

**Table 4:** Cox proportional hazards analysis for 30-day, 90-day, and one-year mortality among centenarian trauma patients

	30-day mortality HR (95% CI)	*p*	90-day mortality HR (95% CI)	*p*	1-year mortality HR (95% CI)	*p*
Male sex	0.945 (0.631–1.417)	0.786	1.320 (0.998–1.747)	0.052	1.269 (1.012–1.592)	0.039
Hip fracture	2.962 (2.162–4.057)	< 0.001	2.529 (2.014–3.176)	< 0.001	2.080 (1.750–2.472)	< 0.001
Admission age, years	1.007 (0.969–1.046)	0.732	0.991 (0.961–1.022)	0.567	0.971 (0.945–0.997)	0.027
Healthcare level		< 0.001		0.001		< 0.001
Tertiary vs. Primary	0.020 (0.002–0.163)	< 0.001	0.022 (0.003–0.173)	< 0.001	0.019 (0.002–0.150)	< 0.001
Tertiary vs. Secondary	0.024 (0.003–0.197)	< 0.001	0.023 (0.003–0.181)	< 0.001	0.017 (0.002–0.137)	< 0.001
CIHI-HFRM	0.026 (0.006–0.118)	< 0.001	0.091 (0.031–0.271)	< 0.001	0.084 (0.036–0.195)	< 0.001
CIHI-HFRM (per 0.1 increase; CIHI×10)	0.694 (0.600–0.808)	< 0.001	0.787 (0.707–0.878)	< 0.001	0.803 (0.740–0.872)	< 0.001
CIHI-HFRM (per 1 deficit; CIHI×36)	0.904 (0.868–0.942)	< 0.001	0.936 (0.908–0.964)	< 0.001	0.941 (0.920–0.963)	< 0.001
mFI-5	0.782 (0.683–0.896)	< 0.001	0.899 (0.816–0.990)	0.031	0.906 (0.840–0.976)	0.010
Hip fracture	2.847 (2.081–3.896)	< 0.001	2.439 (1.945–3.060)	< 0.001	1.992 (1.678–2.365)	< 0.001
Male sex	0.978 (0.652–1.466)	0.915	1.363 (1.030–1.803)	0.030	1.301 (1.037–1.633)	0.023
Admission age, years	1.021 (0.978–1.066)	0.342	0.996 (0.961–1.031)	0.809	0.969 (0.941–0.998)	0.034
Healthcare level		< 0.001		0.001		< 0.001
Tertiary vs. Primary	0.016 (0.002–0.127)	< 0.001	0.018 (0.002–0.142)	< 0.001	0.015 (0.002–0.117)	< 0.001
Tertiary vs. Secondary	0.019 (0.002–0.152)	< 0.001	0.018 (0.002–0.146)	< 0.001	0.013 (0.002–0.104)	< 0.001

All models were adjusted for sex, hip fracture, admission age (years), and healthcare facility level. Healthcare facility level was entered as a categorical variable, with tertiary care as the reference category; “Healthcare level (overall)” reports the global p-value for this factor. Frailty was modeled in two ways: The first model used CIHI-HFRM (continuous), calculated as total deficits/36 (range 0–1). HRs are presented for the original CIHI-HFRM scale and for a 0.1 increase (CIHI×10) and 1 additional deficit (CIHI×36) for interpretability. The second model presents a sensitivity analysis replacing CIHI-HFRM with the mFI-5

CIHI-HFRM, Canadian Institute for Health Information Hospital Frailty Risk Measurement; mFI-5, 5-factor modified frailty index; HR, hazard ratio; CI, confidence interval

## Discussion

This national cohort examined orthopedic conditions among centenarians admitted to the emergency department between January 2016 and July 2024, and investigated the relationship between frailty and short- and long-term mortality in this patient group. The most common presentation in this age group was hip fracture, followed by fractures of the upper extremity. The striking finding of our study was that frailty was not a risk factor for mortality at 1, 3, or 6 months, despite being protective according to CIHI-HFRM and mFI-5. This paradoxical finding necessitated a reevaluation of the concept of frailty in centenarians. Moreover, male sex was associated with increased 1-year mortality, while chronological age was related to decreased 1-year mortality. Importantly, the level of the healthcare facility emerged as a strong determinant of survival, with treatment in higher-level centers being associated with markedly lower mortality rates. Finally, the presence of hip fractures was identified as another predictor of mortality.

Interestingly, our results revealed that higher frailty scores, as measured by the CIHI-HFRM and mFI-5, were associated with lower mortality rates in both the short- and long-term. This finding is counterintuitive, since frailty is generally expected to increase vulnerability to adverse outcomes and is a strong predictor of morbidity and mortality, also in trauma [17]. However, the reverse relationship found in centenarians could be explained by “survivorship bias”. Although persons aged 100 years and older have higher frailty scores, they constitute a distinct group that is probably more biologically resilient and genetically advantaged, and has stronger and more supportive social bonds [11]. Various hypotheses and underlying mechanisms have been proposed to explain the longevity of centenarians in the literature. It has been demonstrated that specific genetic variants, such as FOXO3A and APOE ε2, confer protective effects against cardiometabolic and neurodegenerative diseases, thereby contributing to longevity in these individuals [18, 19]. Furthermore, despite the concept of “inflammaging”, it has been suggested that centenarians exhibit low levels of systemic inflammation and a more balanced immune response, which provides resistance against age-related degenerative processes [12, 20]. In addition, lifestyle and environmental factors such as low tobacco and alcohol consumption, healthy eating habits, lifelong physical activity, and strong social connections are also considered important determinants of this long survival [21]. Therefore, even if classified as frail, centenarians who have developed different adaptation mechanisms to various stressors throughout their lives may show better mortality outcomes than expected after trauma due to their biological reserves, genetic advantages, and social support mechanisms.

Another possible explanation for the protective association between frailty and mortality is differential exposure to beta-blocker therapy. Frailer patients with higher cardiovascular morbidity are more likely to receive chronic beta-blockers, which may reduce hyperadrenergic stress after a hip fracture and surgery [22, 23]. Large cohorts, ongoing beta-blocker therapy linked to lower short-term mortality post-surgery, despite users being older with higher risks [24]. It is also associated with better 1-year survival [25]. This could partly explain the decreased mortality in frailer patients, but data on beta-blocker use was unavailable in our dataset, so residual confounding cannot be ruled out.

Another noteworthy finding in our study is that age was a protective factor for 1-year mortality. Similar to frailty, although this situation may seem paradoxical at first glance, it can be explained by biological and methodological factors specific to the centenarian population; the “longevity selection” effect is at play among individuals aged 100 and above [22, 23]. Indeed, in a large cohort study conducted by Andersen and colleagues as part of the New England Centenarian Study, it was reported that the age of onset of diseases, such as cardiovascular disease, cancer, dementia, and stroke, was progressively delayed in individuals aged 100 years and older, and that the phenomenon of “morbidity compression” was observed. It has been reported that, particularly among supercentenarians (≥ 110 years), the frequency of severe functional loss and multimorbidity is low, even in the very advanced stages of life, suggesting that the health span is approaching the life span [4]. This finding suggests that increasing age among centenarians may paradoxically be associated with higher biological resilience and lower mortality. Therefore, the protective effect of age in our study may reflect these individuals’ biological reserves, genetic advantages, and the compression of morbidity into later life.

Our findings are broadly aligned with the existing literature on orthopedic trauma in the centenarian population. However, the relationship between fracture characteristics and mortality has not been uniform across studies. A retrospective study conducted by Gündoğdu et al. reported no significant association between fracture localization and mortality and found that treatment preference did not significantly affect mortality in their cohort [24]. In contrast, in our nationwide analysis, hip fracture emerged as a strong predictor of mortality across follow-up periods. These data support the concept that post-traumatic outcomes in centenarians may be influenced not only by injury patterns but also by strong survival selection at extreme ages, like “biological resilience” and “survivor effect,” and potentially by healthcare factors affecting access and quality of care. When the “compression of morbidity” phenomenon reported by Andersen et al. and the findings of Gündoğdu et al. are considered together, it becomes evident that these individuals experience morbidity later in life and that their post-trauma mortality risk is determined more by factors such as biological resilience [4, 24].

Consistent with the literature, the most common orthopedic injuries observed in centenarians in our study were hip fractures, particularly femoral neck and intertrochanteric fractures. These fractures were followed by upper extremity fractures and fragility fractures resulting from low-energy falls [25]. Also, in our study, hip fracture was the factor that significantly increased the risk of mortality among trauma types. This finding, which has a strong and consistent effect on both 30-day and 1-year mortality, is consistent with prior literature. Roche et al. reported a 1-year mortality rate of 38% after hip fracture in patients aged 100 years and older [26]. A meta-analysis by Lastoria et al. found that one-year mortality following hip fracture in centenarians was approximately 53.8% [27]. Another retrospective review of 60 centenarians with hip fractures reported mortality rates of 27% at 30 days, 40% at 3 months, and 55% at one year [28]. In one of the most recent and largest series, Gündoğdu et al. reported mortality rates of 16.2%, 28.4%, and 41.9% at 1 month, 6 months, and 1 year, respectively [24]. The fact that the 1- and 3-month mortality rates in our series are almost identical to in those studies. Therefore, hip fractures in this age group should be considered not only as a local orthopedic problem but also as a systemic mortality determinant [29, 30].

Furthermore, we found that the level of healthcare is a predictor of mortality in centenarians admitted with trauma. The lower mortality risk observed among centenarians treated at tertiary care centers can be attributed to a multidisciplinary approach, intensive care facilities, experienced surgical teams, and early management of complications. Similarly, in a review of 37 studies with 37,294 participants, orthogeriatric care was associated with lower in-hospital and 1-year mortality [31]. This situation demonstrates that the prognosis after trauma in older persons is closely related not only to individual clinical factors but also to access to the healthcare system and quality of care. Particularly in the centenarian population, early referral to high-level healthcare centers has been shown to have a mortality-reducing effect [32, 33].

We acknowledge that our study has certain limitations. First, due to its retrospective design, data loss and incomplete records are inevitable. Second, frailty assessment was performed using ICD codes rather than direct clinical examination or face-to-face tools; this may not fully capture frailty. Furthermore, patients’ pre-trauma functional status, levels of care support like home care or institutional care, and post-trauma functionality could not be assessed objectively via performance tests. Detailed treatment data, including regularly used medications such as beta-blocker therapy, history of surgery, surgical technique, surgical complications, and postoperative rehabilitation, were not recorded. Because CIHI-HFRM and mFI-5 are ICD-10-based measures with multiple comorbidity categories, we did not adjust for overlapping comorbidities to reduce multicollinearity. Residual confounding by comorbidity and treatment selection cannot be fully excluded, which may contribute to the paradoxical association between frailty and mortality. However, our study also has strengths. First, it presents one of the largest series reported from Turkey regarding orthopedic trauma in the centenarian population. Furthermore, it contributes to the literature by examining not only mortality rates but also possible risk factors associated with mortality. Moreover, both short- and long-term mortality were evaluated, with clinically relevant predictors such as fracture site and facility level. Frailty was measured using an ICD-based index, and the findings were supported by the mFI-5. Finally, the unexpected protective association of age and the paradoxical frailty–mortality pattern help generate new hypotheses regarding survivorship selection and mechanisms of resilience in centenarians.

## Conclusion

Our study is one of the most comprehensive series evaluating the prevalence of orthopedic trauma, mortality rates, and the predictive value of frailty in centenarians. However, findings that do not align with the protective effect of age and the classic relationship between frailty and mortality may be explained by mechanisms such as the biological resilience of centenarians, their genetic advantages, and the fact that their health span approaches their lifespan. The data obtained emphasize the importance of multidisciplinary and personalized approaches in the management of trauma in older adults and serve as a valuable reference for future studies.

## Supplementary Information

Below is the link to the electronic supplementary material.

Supplementary Material 1

Supplementary Material 2

## Data Availability

The data that supports the findings of this study are available on reasonable request from the corresponding author.
