# Supplementary material for: Emergency trauma admissions in the oldest-old: short- and long-term mortality and the role of frailty in a Turkish national cohort of centenarians
Source: Eur J Trauma Emerg Surg. 2026 Mar 9;52(1):88. doi: 10.1007/s00068-026-03137-0 (PMC12971919; doi:10.1007/s00068-026-03137-0)
Supplement: Supplementary file 1 — Supplementary Material 1 [file 68_2026_3137_MOESM1_ESM.docx]

**Supplementary Table S1. ICD-10 code definitions for diagnosis of frailty variables**

| **CIHI-HFRM Variable** | **ICD-10 codes included** |
| --- | --- |
| Anemia | **D64.*** |
| Depression / anxiety / stress-related disorders | **F32.***, **F33.***, **F34.1**, **F38.***, **F40.***, **F41.***, **F43.***, **F44.*** |
| Dysphagia / dysphasia / language disorders | **R13.***, **R47.0**, **F80.1–F80.3**, **G31.01** |
| Arthritis / arthrosis / inflammatory rheumatic disease | **M00–M19**, **M31.5**, **M32–M36.4**, **M45**, **M46.5**, **M46.9** |
| Asthma | **J45.*** |
| Dizziness / vertigo / gait or mobility abnormality | **R42**, **R26.*** |
| Malignancy | **C00–C97**, **D00–D09**, **D10–D36**, **D37–D48** |
| Arrhythmia | **I47.***, **I48.***, **I49.*** |
| Cardiac disease (valvular/endocarditis/cardiomyopathy/myocarditis) | **I05–I09.0**, **I34–I39.***, **I41.2***, **I42.***, **I43.*** |
| Chest pain | **R07.1–R07.4** |
| Cerebrovascular disease / stroke / Transient ischemic attack | **I60–I64**, **I69.***, **G45.*** (includes I63.* subtypes) |
| Chronic obstructive pulmonary disease/ chronic bronchitis / emphysema / bronchiectasis | **J44.***, **J42**, **J43.***, **J47** |
| Heart failure | **I50.0**, **I50.1**, **I50.9** |
| Coronary artery disease | **I25.1** |
| Diabetes mellitus | **E10.***, **E11.***, **E13.***, **E14.*** |
| Delirium | **F05.*** |
| Delusional disorders | **F22**, **F24** |
| Dementia | **G30.***, **F00.***, **F01.***, **F02.***, **F03** |
| Emphysema | **J43.***, **J98.2**, **J98.3**, **T79.7**, **T81.81** |
| Endocrine / metabolic / nutrition / fluid–electrolyte disorders | **E05.***, **E16.***, **E46**, **E53.***, **E55.***, **E83.***, **E86**, **E87.*** |
| Epilepsy | **G40.*** |
| Injury / external cause / procedure-related injury | **X59.***, **Y84.*** |
| Falls | **W00–W19** |
| Weakness / frailty codes | **R53**, **R54** |
| Care dependency / need for assistance | **Z74.0–Z74.9**, **Z75.0–Z75.9**, **Z99.0–Z99.9** |
| Gastrointestinal disorders (as coded) | **A09**, **K26**, **K52**, **K59** |
| Hallucinations | **R44.0–R44.3** |
| Sensory loss (vision/hearing) | **H54.***, **H91.*** |
| Hemiplegia / paraplegia | **G81.***, **G82.*** |
| Hypertension / hypotension | **I10–I13**, **I15.***, **I95.*** |
| Incontinence | **R15**, **R32**, **N39.3**, **N39.4** |
| Infection / colonization | **A04.***, **A41.***, **B96.***, **Z22.*** |
| Mental illness / substance use (as coded) | **F10.***, **F22**, **F24** |
| Mobility/muscle wasting related | **M62.3**, **R26.***, **Z74.*** |
| Scoliosis | **M41.*** |
| Ostomy status | **Z93.*** |
| Pain | **R52.*** |
| Parkinson’s disease | **G20.*** |
| Pressure ulcer | **L89.*** |
| Cachexia | **R64** |

* indicates inclusion of all subcodes under the specified ICD-10 category.
