# Supplementary material for: Emergency trauma admissions in the oldest-old: short- and long-term mortality and the role of frailty in a Turkish national cohort of centenarians
Source: Eur J Trauma Emerg Surg. 2026 Mar 9;52(1):88. doi: 10.1007/s00068-026-03137-0 (PMC12971919; doi:10.1007/s00068-026-03137-0)
Supplement: Supplementary file 2 — Supplementary Material 2 [file 68_2026_3137_MOESM2_ESM.docx]

Supplementary table S2. 1-year mortality across CIHI-HFRM frailty risk groups

| **CIHI Risk Group** | **Survivor 1 year n (%)** | **Non-survivor 1 year n (%)** | **Total n** |
| --- | --- | --- | --- |
| 1 (0–1 deficit) | 9 (39.1) | 14 (60.9) | 23 |
| 2 (2–3 deficits) | 25 (34.7) | 47 (65.3) | 72 |
| 3 (4–5 deficits) | 72 (50.0) | 72 (50.0) | 144 |
| 4 (6–7 deficits) | 85 (45.2) | 103 (54.8) | 188 |
| 5 (8–9 deficits) | 119 (53.1) | 105 (46.9) | 224 |
| 6 (10–12 deficits) | 150 (56.0) | 118 (44.0) | 268 |
| 7 (13–15 deficits) | 116 (69.5) | 51 (30.5) | 167 |
| 8 (≥16 deficits) | 59 (62.8) | 35 (37.2) | 94 |

Chi-square p-value for group differences: <0.001. P for trend across ordered risk groups: <0.001.
